# Signal alterations of the basal ganglia in the differential diagnosis of Parkinson’s disease: a retrospective case-controlled MRI data bank analysis

**DOI:** 10.1186/1471-2377-12-163

**Published:** 2012-12-29

**Authors:** Sarah Jesse, Jan Kassubek, Hans-Peter Müller, Albert C Ludolph, Alexander Unrath

**Affiliations:** 1 Department of Neurology, University of Ulm, Ulm, Germany

**Keywords:** Parkinson’s disease, MRI, Substantia nigra, Globus pallidus internus, Progressive supranuclear palsy, Morphological changes

## Abstract

**Background:**

Based upon the acquainted loss of dopaminergic neurons in the substantia nigra in Parkinson’s disease (PD), we hypothesised changes in magnetic resonance imaging signal intensities of the basal ganglia to be useful as an additional technical tool in the diagnostic work-up.

**Methods:**

Region-of-interest analyses (substantia nigra and globus pallidus internus) of T2-weighted scans were performed in seventy subjects with PD, 170 age- and gender-matched controls and 38 patients with an atypical form of neurodegenerative Parkinsonian syndrome (N = 11 multisystem atrophy, N = 22 progressive supranuclear palsy, N = 5 corticobasal syndrome).

**Results:**

In patients with PD, significant changes in signal intensities within the substantia nigra were observed compared to controls at p < 0.001. For the globus pallidus internus, signal alterations in PD and progressive supranuclear palsy were found to be significant (p < 0.001) if compared to controls. Furthermore, signal changes of substantia nigra correlated with signal intensities of globus pallidus internus in the ipsilateral hemisphere in both groups. Sensitivity was 86% and specificity was 90% for the combined analysis of substantia nigra and globus pallidus internus in the complete patient sample versus controls.

**Conclusions:**

Signal alterations of substantia nigra and globus pallidus internus in routine magnetic resonance imaging were useful to distinguish patients with PD from controls. In addition, signal changes in globus pallidus internus could be used to differentiate progressive supranuclear palsy patients from controls. These analyses have the potential to serve as an additional non-invasive technical tool to support the individual differential diagnosis of PD.

## Background

So far, the diagnosis of Parkinson’s disease (PD) is mainly based on the presence of characteristic features in a set of clinical assessments [1,2]. Nevertheless, about 32% of post mortem proven PD were not identified by those criteria. Jankovic et al. found 65% of those cases who had initially been diagnosed as PD to have an alternate diagnosis after the 7.6-year follow-up [3], including multisystem atrophy (MSA), progressive supranuclear palsy (PSP), Alzheimer’s disease (AD) as well as cerebrovascular events, indicating that the clinically based diagnosis provides limited accuracy [2,4]. Non-motor symptoms are increasingly used for the clinical diagnosis, including hyposmia, sleep disturbances, behavioral/emotional dysfunction, dysautonomia, depressive symptoms, and chronic pain [5]. However, since their sensitivity and specificity cannot be determined yet, it would be beneficial to add further technical tools that might support the clinically suspected diagnoses.

Molecular imaging e.g. with single-photon emission computed tomography or positron emission tomogra-phy represents a diagnostic tool which supports the classification of Parkinsonian syndromes including their early diagnosis in the clinical routine [6]. As a fully non-invasive imaging tool, ultrasound sonography of the substantia nigra (SN) can be used for the investigation of regional hyperechogenicity, but requires an experienced observer for the evaluation [7]. Conventional magnetic resonance imaging (MRI), on the contrary, is only used yet for exclusion of other underlying pathologies, e.g. lesions of vascular origin or MRI findings suspicious for atypical Parkinsonian forms [8]. Several attempts were performed to explore PD-associated brain alterations via MRI like diffusion tensor imaging, and some of those studies revealed promising results with a regional decrease in fractional anisotropy in the SN of PD patients compared with control persons [9-12]. Unfortunately, those advanced MRI-based methods are not applicable in routine diagnostic assessments yet as specific data acquisition and time-consuming post-processing procedures are still essential for evaluation of these approaches.

Thus, we performed an MRI-based study in PD on the basis of the following conceptual assumptions and hypotheses: (i) signal alterations in MRI have to be investigated in routine diagnostics to be easily available and broadly applicable, (ii) we suspected signal changes to be visible in SN as well as the corresponding globus pallidus internus (GPI) [13], (iii) MRI signal intensities of those regions should be able to differentiate at least PD from control subjects (CON) and possibly from atypical Parkinsonian syndroms in order to be useful as an additional technical tool in the diagnostic workup. Accordingly, we investigated whether or not signal changes in MRI correlated with the clinical presentation of symptoms with respect to the more affected side.

## Methods

### Patients

The study was performed as a retrospective case-controlled MRI data bank analysis. MRI data acquisition in patients with Parkinsonian syndromes and controls had been approved by the Ethics Committee of the University of Ulm, Germany, and patients and controls gave informed consent to the scientific use of their anonymised data sets. The study was in compliance with the Helsinki Declaration. Seventy consecutive patients with PD who had been clinically diagnosed by board-certified neurologists on the basis of the UK Brain Bank criteria for the diagnosis of PD [14,15] were included. For patients’ ages and Hoehn and Yahr stages, please see Table 1.

**Table 1:** Overview of clinical data of all patient groups

	**N**	**Age (mean ± SD)**	**Hoehn & Yahr**	**Duration of disease in years (mean ± SD)**
**PD**	70	71 ± 9	stage 1: 2/70	9.0 ± 0
			stage 2: 13/70	6.1 ± 4.8
			stage 3: 22/70	5.0 ± 4.7
			stage 4: 23/70	7.2 ± 5.3
			stage 5: 10/70	7.6 ± 6.8
**MSA**	11	62 ± 6	-	2.4 ± 1.6
**PSP**	22	70 ± 8	-	3.0 ± 2.4
**CBS**	5	65 ± 4	-	2.3 ± 1.9
**CON**	170	66 ± 10	-	-

Abbreviations: *PD* idiopathic Parkinson’s disease, *MSA* multiple system atrophy, *PSP* progressive supranuclear palsy, *CBS* corticobasal syndrome, *CON* control persons.

The additional patient groups with an atypical form of Parkinsonian syndrome comprised 11 subjects with MSA (diagnosed according to [16]), 22 patients with progressive supranuclear palsy (diagnosis was set according to [17]) as well as 5 patients with corticobasal syndrome (diagnosed according to [18]). As the control group, 170 patients without any neurological sign of extrapyramidal motor symptoms were included, the most common diagnoses were transitory ischemic attack (N = 35), dizziness (N = 31), seizures (N = 20), headache (N = 13) and paralysis of the seventh brain nerve (N = 10). All MRI data sets of the controls were thoroughly checked for any structural brain pathology by two independent experienced observers (JK, AU). Additional clinical data for all patients and controls are given in Table 1.

### Data acquisition and post-processing

MRI scans were acquired on a 1.5 Tesla routine clinical MRI scanner (SIEMENS MAGNETOM Symphony, a TIM system, Erlangen, Germany) equipped with a standard 12 channel head coil. As elderly patients often show microvascular lesions, exclusion criteria were used according to [19], only allowing grade 0 or 1, the latter only if not found in the basal ganglia, and the respective data sets of patients with higher-grade vascular lesions were not considered for statistical evaluation.

All patients and controls underwent the same clinical routine MRI protocol, consisting of whole-brain covering sequences of diffusion-weighted imaging (DWI) including apparent diffusion coefficient-maps (ADC), T1-, T2-, T2*-weighted imaging as well as fluid-attenuated inversion recovery (FLAIR). The sequence parameters are given in the Additional file 1: Table S1. In the following, we focused all analyses on T2-weighted image data, all additionally acquired data sets were only used to rule out further brain pathologies.

### ROI analysis

All data analyses were performed by two independent experienced observers. Calculation of test/retest (i.e., intra-rater) and inter-rater reliability was performed measuring of the bilateral SN and GPI ROIs as well as ROIs in the sinus and lateral cerebral ventricles in a subset of 55 patients, including PD and CON. Statistical evaluation was performed according to Bland and Altman [20].

By use of a dedicated certified workstation (AGFA, IMPAX > ES, DS 3000, Belgium), regions of interest (ROI) in the SN as well as the GPI were manually traced by the two specifically trained raters with a mouse driven cursor using T2-weighted axial images. The raters were blinded to the diagnosis. The T2-weighted images allow for the identification of the SN pars reticulata as a hypointense area in the posterior region of the crus cerebri, while a relative hyperintense area between the SN pars reticulata and the red nucleus represents the pars compacta [21,22]. The ROI had the same size in each patient, independent of the existence of hyperintense signal alterations in the SN or the GPI. The ROI size was approximately 2.5 cm × 0.5 cm for SN and 1.5 cm × 0.25 cm for GPI, variability was approximately ± 0.2 cm. T2- weighted imaging provides images with an intensity given in arbitrary units (a.u.) that depends on imaging parameters and calibration parameters of the scanner in addition to internal properties of the tissue. Therefore, a normalisation algorithm was used in which the original histogram of the whole image intensity distribution was stretched and shifted according to defined image intensities [23]:

(1) $$I'\left(x,y\right)=\frac{I_{M}-I_{L}}{I_{\max}-I_{\min}}\left(I\;\left(x,y\right)-I_{\min}\right)\;+I_{L}$$

In Equation 1, *I(x,y)* denotes the original image intensity (averaged in the respective ROI) and *I*_*max*_ and *I*_*min*_ denote normalization intensities which were evaluated from ROIs (positioned in the respective slices) in the cerebrospinal fluid (hyperintense) and in the sinuses (hypointense), respectively. The re-normalized intensity *I’(x,y)* is obtained by re-scaling to *I*_*M*_ and *I*_*L*_ which represent the arithmetic average of intensities of the cerebrospinal fluid (*I*_*M*_) and the sinus (*I*_*L*_), respectively, of all data sets. It should be noted that *I*_*M*_ and *I*_*L*_ define a new intensity histogram whose characteristics can be freely chosen. In the following, the re-normalized intensity *I’(x,y)* is denoted as intensity*.*

### Correlation analysis

We performed correlation analyses of the signal intensities of the SN and the corresponding signal intensities in the GPI of the same side. Since we were interested if the extent of the signal alterations in the SN and the GPI depended on the duration of the disease, these correlation analyses were also performed. In a final step, we compared the MRI findings with clinical symptoms in the PD group to address the question if MRI signal intensities of SN have the potential to predict the clini-cally dominant affected side and vice versa. A step by step explanation of the analysis procedure is given in the Additional file 2: Table S2.

### Statistics

All statistical analyses were performed using the software SigmaStat 3.5 (Systat Software Inc., Chicago, USA). The comparison of signal intensities between the different groups was based on calculations using ANOVA Kruskal Wallis, correlations were calculated by Pearson’s correlation coefficient for comparison of SN and GPI in the same hemisphere. Correlations of signal alterations in SN and GPI with duration of the disease as well as Hoehn & Yahr stages were performed using Spearman correlation. The analyses of test/retest (intra-rater) and inter-rater reliability were performed according to the method by Bland and Altman [20]. In addition, Pearson’s correlation for intra- and inter-rater analysis results was calculated. Statistical significance was set at p < 0.05.

In order to estimate the predictive power of the technique, a threshold for discrimination of signal hyperintensities between the complete patient sample and the control sample was defined. For this purpose, we summarised the complete patient sample including all investigated pathologies, since the low sample sizes of the Parkinsonian syndromes other than PD did not allow for separate analysis. Then, left and right SN values were arithmetically averaged (averaged SN intensity, ASN), the same was performed for left and right GPI values (averaged GPI intensity, AGPI). The threshold for the intensity value for SN was defined as the average value of all ASN of the controls + standard deviation of the controls^′^ ASN values, resulting in a threshold value for ASN (TH_SN_) = 380 arbitrary units (a.u.). In the corresponding way, a threshold for GPI was defined as TH_GPI_ = 310 a.u. Then, pathology was defined in three ways: abnormality for SN (ASN > TH_SN_), abnormality for GPI (AGPI > TH_GPI_), and also a combination, i.e. either ASN or AGPI was higher than the respective threshold (ASN > TH_SN_ or AGPI > TH_SN_). The leave one out method was performed to calculate the predicted residual error sum of squares (PRESS).

## Results

### Signal intensities of SN and GPI

**Figure 1:**
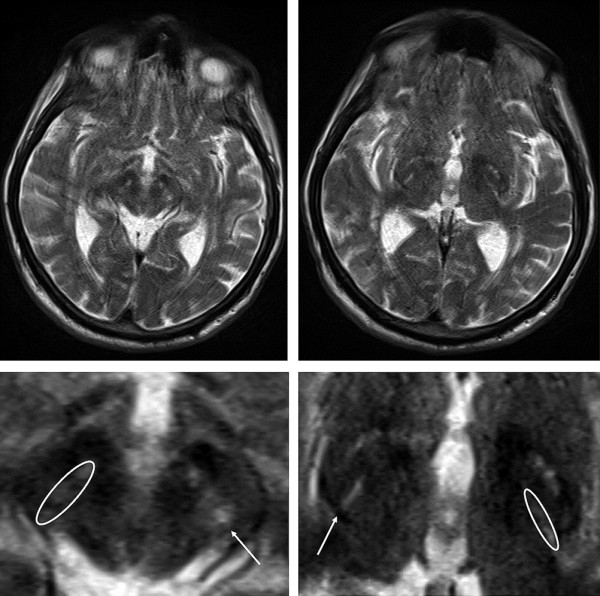
**Illustration of ROI definition in the SN and the GPI.** On the left side, whole brain image at the location of the SN and the magnification of the midbrain with the hyperintense signal of SN are depicted and marked by an arrow. On the right, whole brain image at the location of the GPI and the magnification of the basal ganglia with the hyperintense signal of the dorsomedial GPI are illustrated. Abbreviations: SN = substantia nigra, GPI = globus pallidus internus.

**Figure 2:**
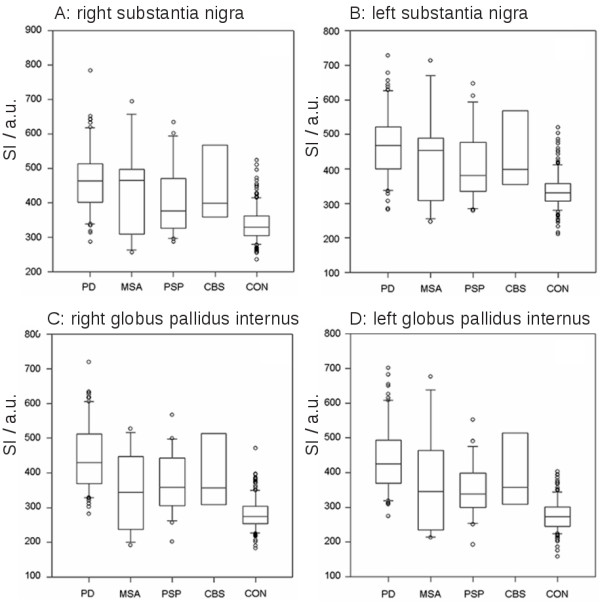
**Signal intensities of the substantia nigra (SN) and the globus pallidus internus (GPI) in the different groups given in arbitrary units (a.u.).** Plot shows 10th, 25th, 50th, 75th, and 90th percentiles and outliers. **A**: Signal intensities of the right SN. **B**: Signal intensities of the left SN. **C**: Signal intensities of the right GPI. **D**: Signal intensities of the left GPI. The comparison between PD and CON was significant for SN on both sides at p < 0.001. For the GPI on both sides, the statistical comparison between PD and CON as well as between PSP and CON showed differences at p < 0.001. Abbreviations: PD = Parkinson’s disease, MSA = multisystem atrophy, PSP = progressive supranuclear palsy, CBS = corticobasal syndrome, CON = control persons.

ROI analyses of the signal intensities of SN and GPI of both sides were performed in the same way for all patients and controls. An example for the ROI-based measurement of signal alterations is demonstrated in Figure 1. In PD patients, we observed hyperintense alterations of the SN, and the comparisons of the corrected signal intensities between PD and CON were significant for the SN in both hemispheres at p < 0.001. In addition, we also observed PD-associated hyperintense signal alterations of the GPI in both hemispheres. To a lesser degree, hyperintense signal alterations of the GPI were observed in PSP patients compared to controls. The statistical comparison of the GPI signal intensities between PD as well as PSP and CON was also significant at p < 0.001. The results of the data analysis are summarised in Figure 2. No significant differences could be found for MSA and CBS compared with CON for either GPI and SN in both hemispheres.

### Comparison of signal intensities of SN and the ipsilateral GPI

**Figure 3:**
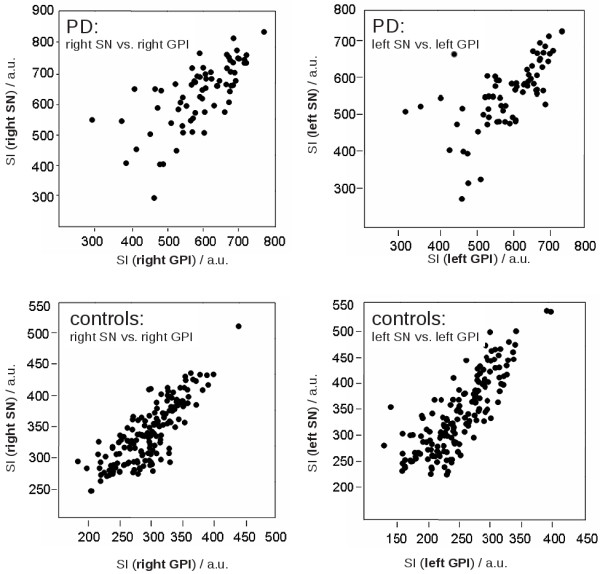
**Correlations of signal intensity (SI) (in arbitrary units a.u.) of substantia nigra with globus pallidus internus on the same side for all groups.** Pearson correlation coefficient was significant for PD, PSP and CON with p < 0.05. Abbreviations: SN = substantia nigra, GPI = globus pallidus internus, for the other abbreviations see Figure 2.

Correlation analyses of the altered signal intensities of the SN and the corresponding signal intensities in the ipsilateral GPI revealed statistically significant correlations for PD as well as for PSP and CON with p < 0.05. Data are depicted in Figure 3.

### Test/retest (intra-rater) and inter-rater reliability

**Figure 4:**
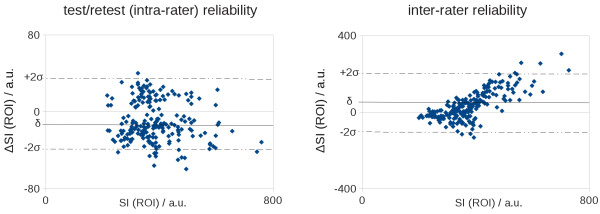
**Test/retest and interrater reliability according to Bland-Altman. Horizontal lines indicate δ (bias – straight line) and the 2σ intervall (δ ± 2σ, σ - standard deviation – dashed line, limit of accordance). A**: test/retest (intra-rater) reliability of 94% is within the limit of accordance. **B**: inter-rater reliability of 96% is within the limit of accordance. SI (ROI) is the average signal intensity within a ROI in a.u., ΔSI (ROI) is the test difference in a.u.

Here, the scatter of differences of single pair-values was compared. In the case of a symmetrical distribution of differences, 95% of the values are located in the range of δ ± 2σ (δ, bias; σ, standard deviation). This limit of accordance is represented as dashed lines in Figure 4. For test/retest reliability (intra-rater reliability), 94 % of tests were within the 2σ range representing an acceptable reproducibility of the test procedure. Also for inter-rater reliability (two raters), 96% of tests were within the 2σ range, indicating a good reproducibility of data analysis. Correlations for intra-rater variability and inter-rater variability were 0.97 (p < 0.0001) and 0.62 (p < 0.0001), respectively.

### Comparison of signal intensities in SN and GPI with duration of the disease and H&Y stages

Correlation analyses of the extent of signal alterations in the SN and the GPI and the duration of the disease showed no significant changes (p > 0.05); as a limiting factor, duration of the disease could not be determined retrospectively for 9 out of 70 PD patients. The comparison of alterations in both anatomical areas with Hoehn & Yahr stages showed a significant positive correlation at p < 0.03.

### Comparison of clinical symptoms with the MRI data

Here, positive predictive values, analysed separately for both sides, were not promising as their levels were in random distribution (positive predictive value (PPV) for the left side of SN ([17/(17 + 15)] = 0.53; PPV for the right side of SN [13/(13 + 12)] = 0.52).

### Calculation of sensitivity, specificity, positive predictive value, and negative predictive value

Statistical measures (sensitivity, specificity, PPV, NPV as well as statistical data of the leave one out method) are given in Table 2. In summary, the thresholds calculated as described above (380 a.u. for SN and 310 a.u. for GPI, respectively) showed a high sensitivity (86%) and NPV (90%) for the combination of SN and GPI analysis in the complete patient sample versus controls. Specificity and PPV were similar for SN and GPI investigations, while sensitivity and NPV were lower for SN than for GPI.

**Table 2:** Statistical calculation of sensitivity, specificity, positive predictive value, and negative predictive value (including leave one out method)

	**Sensitivity**	**Specificity**	**PPV**	**NPV**
ASN > TH_SN_	0.72 ± 0.08	0.82 ± 0.02	0.72 ± 0.04	0.82 ± 0.06
AGPI > TH_GPI_	0.84 ± 0.06	0.80 ± 0.03	0.73 ± 0.04	0.89 ± 0.04
Combination	0.86 ± 0.04	0.77 ± 0.05	0.70 ± 0.06	0.90 ± 0.04

Table 2 Statistical measures for defined thresholds: average substantia nigra (ASN) value above threshold (THSN), average globus pallidus internus (AGPI) above threshold (THGPI), and the combination, i.e. ASN > THSN or AGPI > THGPI. Predicted residual error sum of squares (PRESS) values were calculated by the leave one out method. Results are presented as statistical measure ± PRESS. *PPV* positive predictive value, *NPV* negative predictive value.

## Discussion

Since PD diagnosis is still mainly set on clinical assessments based upon the presence of characteristic features, an additional technical tool to support the clinically suspected diagnosis would be beneficial. Unfortunately, broadly available non-invasive neuroimaging tools are limited mainly to transcranial sonography which crucially depends on the rater’s expertise, whereas MRI is primarily performed to exclude other underlying pathologies.

For these reasons, we investigated MRI signal intensities of SN and GPI in a clinical routine setting using a clinical 1.5T MRI scanner with a standard protocol for acquisition and a simple ROI-based post-processing. We observed T2-hyperintense signal changes of both the SN and the GPI areas to be useful to support the diagnosis of PD, whereas controls did not show these features. Additionally, signal intensities of SN correlated with the GPI area of the same side. In addition to these observations in PD and consistent with previous MRI-based studies in atypical Parkinsonian syndromes, signal changes in GPI were confirmed which can also be potentially used to differentiate PSP patients from controls [24]. In order to serve as an additional technical tool in the diagnosis of PSP, these data have to be reproduced and validated using a dedicated study design with investigation of larger numbers of PSP patients.

The thresholds (using the “or” combination) allowed for a high sensitivity (86%) and NPV (90%) for the sample of all investigated pathologies. (Although MSA and CBS showed no significant differences in the comparison with the control group, most probably due to the low sample size, the complete patient group was compared with controls in order to encompass all investigated differential diagnoses.) Specificity and PPV were similar for SN and GPI investigations, while sensitivity and NPV were lower for SN than for GPI. The combined analysis showed similar statistical values for sensitivity and NPV as GPI alone. Taken together, we suggest the use of a combination of GPI and SN in order to consider supplementary information of SN rather than investigating GPI alone.

Our results are in general agreement with previous studies focusing on conventional MRI-based techniques in the diagnosis and differential diagnosis of PD, which revealed a broad spectrum of signal alterations in T1-, T2- or T2*-weighted imaging within the SN [8,25]. In contrast to most previous studies, however, we did not investigate iron or hemosiderin depletion within the SN, visualised by an increase of the T2 and T2* decay resulting in selective shortening of these relaxation times, but rather T2-hyperintensities as a correlate of selective and focal gliosis illustrating the upmost relevant neurodegenerative component of PD. In this context, our results partly reflect the well-known neuroanatomical projectional system of dopaminergic neurons of the SN to the GPI without the necessity of advanced imaging techniques such as tractography [26]. Beyond that, the presented altered signal intensities within the ROIs in the deep brain structures were derived on an individual basis without the need for any post-processing procedures.

What may be the pathophysiological correlate of the signal alterations observed in the SN and GPI of our PD patients? According to Braak et al., pathophysiological processes of PD can be divided into six stages each of which is marked by the continuous development of distinctive inclusion Lewy-bodies and evolves sequentially with the beginning at definite predisposed vulnerable sites, advancing from there in a predictable form through the grey matter of the brain [27]. Remarkably, the course of the disease can clinically be divided into a premotor (stages 1–3) and a symptomatic phase (stages 4–6), while the SN and the basal ganglia are pathophysiologically not impaired in the premotor stages. In short, in Braak stage 3, the continuous, self-propagating process reaches the mid- and forebrain including the pars compacta of the SN, followed by the anteromedial temporal mesocortex, the basal ganglia, the limbic system as well as the prefrontal cortex in stage 4 while in the last two stages, pathoanatomical involvement includes the association areas of the neocortex [1,13,28-31]. On the basis of this knowledge, hyperintensities of SN and GPI, as observed in our MRI data, may be a morphological sign of neurodegenerative processes in the SN with consecutive degeneration of projection fibers to the corresponding GPI. This assumption may also be strengthened by recent reports on stage-dependent SN signal reduction as a putative marker of neuromelanin loss in PD patients in high-resolution T1-weighted imaging with magnetization transfer effect at 3 Tesla [32]. As an alternative theory, one could postulate local magnetic field disturbances as a correlate of iron accumulation in the SN, but we do not favour this hypothesis since T2*-weighted images did not show any substantial signal alterations of the SN in our sample that should be the case in iron deposition (data not shown). However, this lack of evidence for iron deposit might not only be a bias effect in our patient sample, but could also be related to the applied T2*-sequence parameters and also the field strength of our scanner. Here, recent studies report a traceable increase in susceptibility of the pars compacta in PD patients compared with controls by use of susceptibility mapping at ultrahigh field (7 Tesla) and advanced post processing methods [33].

There are some additional limitations to the interpretation of these results. The major limitation is the application of the MRI data analysis to a sample of clinically diagnosed patients in a retrospective design. However, due to the explorative approach of this pilot study, the design with inclusion of clinically diagnosed patients in different (including advanced) clinical stages was chosen. Due to its design, the study cannot overcome the challenge of missed PD diagnosis since it aimed to provide imaging markers for identification of PD patients using clinical assessment as the gold standard rather than pathology. In the future, in order to test the value for early PD diagnosis or differential diagnostics, a prospective design in early affected patients or cases of clinical doubt or a retrospective design comprising patients with neuropathologically proven PD diagnosis has to be used. In addition, it has to be considered that a possible shortcoming may be the use of the 1.5T MRI scanner which implies a limited local resolution in comparison to higher field strengths although a 12-channel head coil was applied. With respect to data analysis, computerised analysis is necessary instead of pure visual inspection - however, the ROI-based processing can be performed within a few minutes on a standard workstation and does not require any advanced qualification beyond neuroanatomical identification of the basal ganglia structures. In addition, the thresholds used for the calculation of sensitivity and specificity were defined ex post.

In summary, although the demonstrated results are based on clinically diagnosed patients and therefore might include a selection bias, our MRI-based findings seem to have the potential to serve as an additional non-invasive neuroimaging-based technical tool within the diagnostic work-up for individual diagnosis of PD. Future studies should set focus on the potential of this ROI-based MRI analysis in early PD patients and might include further technical assessments of the patients such as other imaging modalities including dopamine transporter (DaT) scan. Furthermore, future studies might address the item of the comparison of different computerised MRI-based techniques in the imaging assessment of Parkinsonian syndromes including susceptibility mapping and the analysis of mean diffusivity values in DWI data.

## Conclusions

Addressing the hypothesis-driven rationale of the study, it can be concluded: In PD patients, we suspected signal changes in MRI to be visible in SN as well as the corresponding GPI. This could be confirmed as we found signal alterations in the above mentioned areas in PD patients. Furthermore, MRI signal intensities of those regions should be able to differentiate at least PD from control subjects and possibly from atypical Parkinsonian syndroms in order to be useful as a technical approach to support clinical diagnostic aspects. The examination should serve as an additional diagnostic-supporting tool that could be easily available and broadly applicable using standard routine MRI. This requirement was met using a standard protocol for acquisition and a simple ROI-based post-processing which can be performed within few minutes.

## Competing interests

The authors declare that they have no financial or non-financial conflicts of interests.

## Authors’ contributions

SJ contributed to the acquisition of data, the statistical analysis and drafted the manuscript. JK has made substantial contributions to conception and design and the interpretation of the data. Furthermore, he has been involved in drafting the manuscript and revising it critically for important intellectual content. HPM has made substantial contributions to the analysis (MRI data processing) and the interpretation of the data. Furthermore, he has been involved in drafting the revised manuscript. ACL has made contributions to conception and design of the study. AU has made substantial contributions to conception and design. Additionally, he has given final approval of the version to be published and read the manuscript carefully in view of statistical and MRI-based data. All authors read and approved the final manuscript.

## Pre-publication history

The pre-publication history for this paper can be accessed here:

http://www.biomedcentral.com/1471-2377/12/163/prepub

## Supplementary Material

Additional file 1 **Table S1.** Applied sequence parameters of DWI, T2*w, T2w and FLAIR (scanner: SIEMENS MAGNETOM Symphony, a TIM system, MR B17). Click here for file

Additional file 2 **Table S2.** Sequence of data analysis steps. Click here for file
