# Supplementary material for: Signal alterations of the basal ganglia in the differential diagnosis of Parkinson’s disease: a retrospective case-controlled MRI data bank analysis
Source: BMC Neurol. 2012 Dec 29;12:163. doi: 10.1186/1471-2377-12-163 (PMC3543204; doi:10.1186/1471-2377-12-163)
Supplement: Additional file 1 — Table S1. Applied sequence parameters of DWI, T2*w, T2w and FLAIR (scanner: SIEMENS MAGNETOM Symphony, a TIM system, MR B17). [file 1471-2377-12-163-S1.doc]

**Signal alterations of the basal ganglia in the differential diagnosis of Parkinson’s disease: a retrospective case-controlled MRI data bank analysis.**

Sarah Jesse^1^, Jan Kassubek^1^, Albert C. Ludolph^1^, Alexander Unrath^1^

^1^ University of Ulm, Department of Neurology, Germany

Corresponding author:

Prof. Dr. Jan Kassubek

Department of Neurology

University of Ulm

Oberer Eselsberg 45

89081 Ulm

phone: +49-(0)731-177 1206

email: jan.kassubek@uni-ulm.de

**Supplement table 1:**

Applied sequence parameters of DWI, T2*w, T2w and FLAIR (scanner: SIEMENS MAGNETOM Symphony, a TIM system, MR B17).

| sequence | TOA | PAT | PAT mode | NOS | orientation | FOV in mm | TOS in mm | TR  in ms | TE  in ms | res | TI  in ms | int | FA  in degree | b2-value in s/mm^2^ | BW  in Hz/Px | TF |
| --- | --- | --- | --- | --- | --- | --- | --- | --- | --- | --- | --- | --- | --- | --- | --- | --- |
| DWI | 1:11 | 2 | GRAPPA | 24 | Trans | 230 | 5 | 4600 | 100 | 180 | - | jes | - | 1000 | 958 | - |
| T2*w | 3:13 | 2 | GRAPPA | 24 | Trans | 230 | 5 | 904 | 24 | 256 | - | jes | 30 | - | 90 | - |
| T2w | 2:00 | 2 | GRAPPA | 24 | Trans | 230 | 5 | 5620 | 102 | 448 | - | jes | 150 | - | 130 | 12 |
| FLAIR | 5:00 | none | - | 49 | Coronar | 230 | 3 | 7850 | 112 | 256 | 2300 | jes | 150 | - | 190 | 13 |
| T1w | 3:07 | Fix | - | 24 | Trans | 230 | 5 | 573 | 12 | 86 | - | jes | 70 | - | 130 | - |

Abbreviations: DWI = diffusion weigthed imaging; FLAIR = fluid attenuated inversion recovery; TOA = time of acquisition; PAT = parallel acquisition technique, NOS = number of sheets; FOV = field of view; TOS = tickness of sheets; TR = time of relaxation; TE = time of echo; res = resolution; TI = time of inversion; int = interleaved; FA = flip angle; BW = band width; TF = turbo factor
