# Supplementary material for: Signal alterations of the basal ganglia in the differential diagnosis of Parkinson’s disease: a retrospective case-controlled MRI data bank analysis
Source: BMC Neurol. 2012 Dec 29;12:163. doi: 10.1186/1471-2377-12-163 (PMC3543204; doi:10.1186/1471-2377-12-163)
Supplement: Additional file 2 — Table S2. Sequence of data analysis steps. [file 1471-2377-12-163-S2.doc]

**Signal alterations of the basal ganglia in the differential diagnosis of Parkinson’s disease: a retrospective case-controlled MRI data bank analysis.**

Sarah Jesse^1^, Jan Kassubek^1^, Hans-Peter Müller^1^, Albert C. Ludolph^1^, Alexander Unrath^1^

^1^ University of Ulm, Department of Neurology, Germany

Corresponding author:

Prof. Dr. Jan Kassubek

Department of Neurology

University of Ulm

Oberer Eselsberg 45

89081 Ulm

phone: +49-(0)731-177 1206

email: jan.kassubek@uni-ulm.de

**Additional Table S2:**

Sequence of data analysis steps:

1. T2w images: ROI in the sinus at the same slice as the substantia nigra
2. T2w images: ROI in the substantia nigra, both hemispheres
3. T2w images: ROI in the posterior horn of the lateral ventricles
4. T2w images: ROI in the globus pallidus internus, both hemispheres
5. Calculation of signal intensities [Loizou et al., 2011]
6. Comparison of signal intensities (substantia nigra of both sides among the different groups and globus pallidus internus of both sides among the different groups), using ANOVA Kruskall Wallis
7. Calculation of correlations (Pearson’s correlation coefficient) for the comparison of SN and GPI in the same hemisphere
